# HIV reservoir quantification by five-target multiplex droplet digital PCR

**DOI:** 10.1016/j.xpro.2021.100885

**Published:** 2021-10-11

**Authors:** Claire N. Levy, Sean M. Hughes, Pavitra Roychoudhury, Chelsea Amstuz, Haiying Zhu, Meei-Li Huang, Dara A. Lehman, Keith R. Jerome, Florian Hladik

**Affiliations:** 1 Department of Obstetrics & Gynecology, University of Washington, Seattle, WA 98109, USA; 2 Vaccine and Infectious Disease Division, Fred Hutchinson Cancer Research Center, Seattle, WA 98109, USA; 3 Department of Laboratory Medicine and Pathology, University of Washington, Seattle, WA 98109, USA; 4 Division of Human Biology, Fred Hutchinson Cancer Research Center, Seattle, WA 98109, USA; 5 Department of Global Health, University of Washington, Seattle, WA 98109, USA; 6 Department of Medicine, University of Washington, Seattle, WA 98109, USA

**Keywords:** Bioinformatics, Immunology, Microbiology, Molecular Biology

## Abstract

Most latent human immunodeficiency virus (HIV) proviruses are defective and cannot produce infectious virions. Thus, the number of HIV proviruses with intact genomes is a relevant clinical parameter to assess therapies for HIV cure. We describe high-molecular-weight DNA isolation, followed by restriction enzyme fragmentation that limits cutting within the HIV genome. Multiplexed droplet digital PCR quantifies five targets spanning the HIV genome to estimate potentially intact proviral copies. A reference assay counts the number of T lymphocytes and assesses the level of DNA shearing.

For complete details on the use and execution of this protocol, please refer to Levy et al. (2021).

## Highlights

• Extracting genomic DNA is necessary to determine intactness of HIV-1 provirus
• Selective DNA fragmentation allows droplet formation and maintains proviral intactness
• Results from QuantaSoft™ are analyzed using R scripts or a spreadsheet program
• Final result is intact proviruses per 10^6^ T cells, with shearing correction

## Graphical abstract

**Figure:**
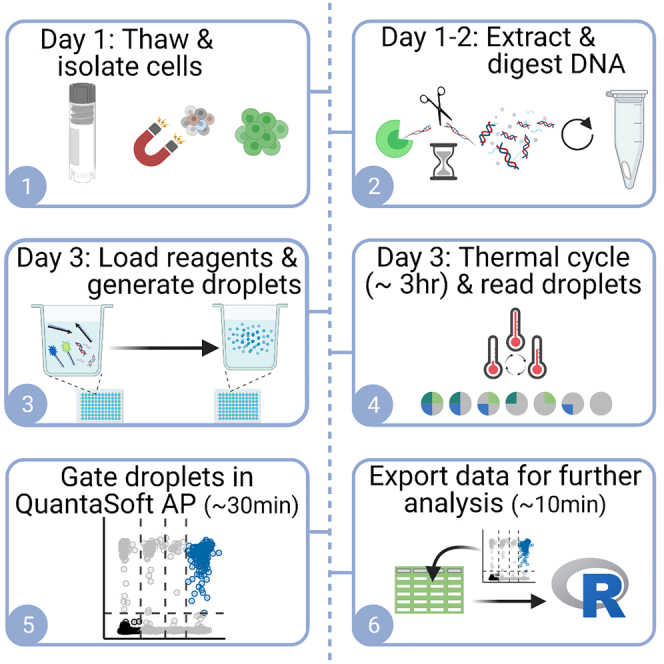

## Before you begin

The protocol below describes the steps for using total peripheral blood mononuclear cells (PBMC), isolated T cells and mucosal tissues. We recommend using samples that are fresh or have either been cryopreserved (tissue or cells) or dry frozen (tissues). This protocol takes 5–8 h of hands-on time spread over 3 days for cell samples or 4 days for tissue samples, due to multiple overnight incubation steps. The instructions for tissues have been tested on mucosal tissues only (specifically, ectocervix, vagina, rectum). Optimization is likely required for other tissue types. The Jurkat and J-Lat 8.4 cell lines may be used for negative and positive controls respectively. Details about these cell lines can be found in the key resources table. In addition to assays targeting regions in the HIV genome, this protocol employs a reference assay to count total cells (using targets in the *RPP30* gene) and all non-T cells (the “deltaD” assay). Primer and probe sequences are listed in Table 1 (HIV targets), Table 2 (target in *TRD*) and Table 3 (*RPP30* targets).

We recommend that the HIV assays be run in triplicate and the reference assay be run in duplicate wells.

Institutional Review Board (IRB) approval to work with human tissues may be needed prior to this experiment. Refer to your institution’s regulations and ensure that all documents are complete before collecting samples or data.

### Prepare HIV and reference gene droplet digital polymerase chain reaction (ddPCR) reagents

1. Prepare lyophilized HIV and deltaD primers and probes.
  a. Resuspend lyophilized primers to 332 μM in Tris-EDTA (10 mM Tris, 0.1 mM EDTA pH 8.0).
  b. Bring all probes to 100 μM in Tris-EDTA (10 mM Tris, 0.1 mM EDTA pH 8.0).
2. Prepare working stocks of HIV gene and deltaD primers/probes (separately for each primer and probe).

**Table:** Primer and probe working stock

Reagent	Final concentration	Amount
ddH_2_O	n/a	85 μL
Fwd primer	16.6 μM	5 μL
Rev primer	16.6 μM	5 μL
Probe	5 μM	5 μL
**Total**	**n/a**	**100 μL**

Store at −20°C for two years

3. Prepare 20**×** stock of the two *RPP30* PrimeTime® Std qPCR Assays by adding Tris-EDTA (10 mM Tris, 0.1 mM EDTA pH 8.0) according to the documentation provided by IDT.
4. Prepare HIV triplex **assay1** pool from the working stocks prepared in step 2 above.
  a. Prepare aliquots and store at −20°C for future use.

***Note:*** Consider the number of aliquots you will need to complete a series of experiments to avoid needing to make a new batch partway through a project.

**Table:** Assay1 pool, amount is for one reaction

Reagent	Final concentration	Amount
*tat* FAM working stock	2.25 μM	1.35 μL
3′*pol* FAM working stock	2.25 μM	0.95 μL
*env* HEX working stock	2.25 μM	0.7 μL
**Total**	**n/a**	**3 μL**

Store at −20°C for two years

5. Prepare HIV triplex **assay2** pool from working stocks prepared in step 2 above
  a. Note that water is added to this master mix simply to make the volume the same as assay1 so that the same volume of master mix can be used for both assays.
  b. Aliquot and store for future use.

**Table:** Assay2 pool, amount is for one reaction

Reagent	Final concentration	Amount
5′*pol* FAM working stock	1.83 μM	1.1 μL
*gag* HEX working stock	0.97 μM	0.55
*env* HEX working stock	1.58 μM	0.95
ddH_2_O	n/a	0.4
**Total**	**n/a**	**3 μL**

Store at −20°C for two years

6. Prepare RPP30_deltaD pool from working stock prepared in step 2 above.
  a. Aliquot and store for future use.

**Table:** RPP30_deltaD pool, amount is for one reaction

Reagent	Final concentration	Amount
5′*RPP30* FAM	2.25 μM	1.35 μL
deltaD FAM	2.25 μM	0.95 μL
3′ *RPP30* HEX	2.25 μM	0.7 μL
**Total**	**n/a**	**3 μL**

Store at −20°C for two years

### Prepare plasmid controls

***Note:*** Please see the Figure S1 for an illustration of how these controls are prepared.

7. Order agar stabs of plasmids from Addgene (see Catalog numbers in key resources table). Grow cultures and extract DNA.
8. Linearize plasmid controls with restriction enzyme *Aat*II. This will result in better droplet formation than circular plasmid. Dilute plasmid such that there will be about 10,000 copies per ddPCR reaction. Calculate the number of copies using the following formula, where *ngSample* is the mass in ng of plasmid that you have and *bpSample* is the length in base pairs of the plasmid:

$$plasmid\;molecules=\left(ngSample\;\times \frac{6.022\times 10^{23}\;molecules}{mole}\right)÷\left(bpSample\;\times \frac{660g\;\text{DNA}}{mole}\;\times \frac{10^{9\;}ng}{g}\right)$$

Combine the linearized plasmids by adding an equal mass of each to a tube.

9. Dilute the mixture with water to 0.01 pg/mL by serial dilution. Save the dilutions at −20°C for future use.
  a. The exact serial dilution plan will depend on the starting stock concentration. We started with a concentration of about 19 ng/μL and performed 3 serial dilutions of 1:100 and a final 1:2 dilution to bring the concentration to approximately 0.1 pg/μL.
10. Prepare plasmid control “P1”: Plasmid control “P1” is a mixture of 7 plasmids and HIV negative human gDNA. Please see the Addgene entries for additional information about the plasmids. Combine the diluted plasmid with *Bgl*I-digested HIV negative human gDNA (see prepare negative control template section below) in a ratio of 4 parts plasmid to 1 part human DNA.
  a. Example: Linearized diluted plasmid is at about 2,500 copies per μL. Combine 4 μL of the plasmid (= 10,000 copies) with 1 μL of *Bgl*I-digested HIV-negative gDNA at a concentration between 100–200 ng/ μL.
  b. Prepare 12 μL aliquots of the P1 control and store at −20°C.
11. For plasmid control “P2”, repeat the steps above but instead of combining the 7 plasmids, **only** use the plasmid that contains all HIV targets: 167347, Seattle_IPDA_control_1_001 (see key resources table).
12. In ddPCR experiments, use 5 μL of P1 or P2 control per control well in assay1 and assay 2. Use 5 ul of 1:2 diluted plasmid P1 or P2 per control well for the RPP30_deltaD reference assay.

***Note:*** Plasmid control signal may vary between preparations and therefore require some optimization. The authors suggest testing a few dilutions of the controls when a new dilution is prepared (for example, neat, 1:5, 1:10).

### Prepare workspace and materials

**Timing: 20 min**

13. Designate a workspace free of plasmid or PCR product contamination. For thawing cells and tissues, work in a biosafety cabinet following your institution’s environmental health and safety protocols for working with HIV-infected human cells or tissues.
14. Set heat block to 56°C
15. Guanidine HCl and guanidine isothiocyanate waste may require special chemical waste disposal depending on your facility’s regulations. Prepare labeled waste containers for liquid and solid waste.
16. If digesting tissues: dissolve any precipitates in buffer ATL by heating to 70°C with gentle agitation.
17. Guanidine HCl, guanidine isothiocyanate and proteinase K should be at room temperature, about 25°C.

### Prepare negative control template

Commercially prepared HIV-negative gDNA can be used as a negative control. Prior to use, this DNA should be digested in *Bgl*I and precipitated with ethanol for later use.

18. Make the following master mix to digest ∼ 20 ug of gDNA:

**Table:** Restriction enzyme digestion of HIV-negative gDNA

Reagent	Final concentration	Amount
NEBuffer 3.1	1**×**	20 μL
NEB *Bgl*I 10,000 IU/mL	750 IU/mL	15 μL
Human HIV-negative gDNA	0.1 μg/mL	20 μg
Molecular grade H_2_O	n/a	To 200 μL
**Total**	**n/a**	**200 μL**

Do not store, begin digestion after preparation

19. Digest at 37°C for 1 h.
20. After digestion, add 100 μL molecular grade H_2_O to bring the total volume to 300 μL, then proceed with EtOH precipitation (Green and Sambrook, 2012) as described below.

## Key resources table

**Table:** 

REAGENT or RESOURCE	SOURCE	IDENTIFIER
**Biological samples**		

Human HIV-negative genomic DNA	Promega	Cat#G3041

**Chemicals, peptides, and recombinant proteins**		

Buffer ATL	QIAGEN	Cat#19076
Buffer EB	QIAGEN	Cat#19086
Guanidine HCl	Research Products International Corp.	Cat#G49000–100.0 CAS No. 50-01-1
3M Sodium acetate	Invitrogen	Cat#AM9740 CAS No. 127-09-3
Guanidine isothiocyanate	Chem Cruz: Santa Cruz Biotechnology Inc.	Cat#SC-202638 CAS No. 593-84-0
*Bgl*I	New England Biolabs	Cat#R0143S
Proteinase K, 20 mg/mL, >318 mAu/mL at 30°C	QIAGEN	Cat#19131
*Aat*II	New England Biolabs	Cat#R0117S
Pen Strep	Gibco	Cat#15140122
RPMI 1640 Medium, HEPES	Gibco	Cat#22400089
Fetal Bovine Serum	Nucleus Biologics	Cat#FBS1824-001
L-Glutamine (200 mM)	Gibco	Cat#A2916801

**Critical commercial assays**		

ddPCR Supermix for Probes (no dUTP)	Bio-Rad	Cat#1863024

**Experimental models: Cell lines**		

J-Lat 8.4 cell line	NIH HIV Reagent Program	Cat#9847-427 RRID:CVCL_8284
Jurkat E6-1 cell line	ATCC	Cat#TIB-152 RRID:CVCL_0367

**Oligonucleotides**		

HIV-1 primers/probes	Integrated DNA Technologies	Table 1
*TRD* gene primers/probes (Zoutman et al., 2017)	Integrated DNA Technologies	Table 2
*RPP30* gene primers/probes	Integrated DNA Technologies	Table 3

**Recombinant DNA**		

Seattle_IPDA_control_1_001	Addgene	Cat#167347
Seattle_IPDA_control_6_002	Addgene	Cat#167348
Seattle_IPDA_control_9_003	Addgene	Cat#167349
Seattle_IPDA_control_14_004	Addgene	Cat#167350
Seattle_IPDA_control_17_005	Addgene	Cat#167351
Seattle_IPDA_control_29_006	Addgene	Cat#167352
Seattle_IPDA_control_32_007	Addgene	Cat#167353

**Software and algorithms**		

Microsoft Office	https://www.microsoft.com/en-us/microsoft-365	N/A
Bioconductor	http://www.bioconductor.org	RRID:SCR_006442
Biorender	http://biorender.io/	N/A
R software 4.0	http://www.r-project.org/	RRID:SCR_001905
QuantaSoft AP 1.0.596	Bio-Rad	http://www.bio-rad.com/en-us/product/qx200-droplet-digital-pcr-system

**Other**		

twin.tec PCR plate 96, semi-skirted	Eppendorf	Cat#951020362
PCR Plate Heat Seal, foil, pierceable	Bio-Rad	Cat#1814040
Bio-Rad PX1 PCR Plate Sealer	Bio-Rad	Cat#1814000
Wide bore p1000 pipette tips	Rainin	Cat#30389195
Wide bore p200 pipette tips	Rainin	Cat#30389217v
Automated Droplet Generation Oil	Bio-Rad	Cat#1864110
Droplet Reader Oil	Bio-Rad	Cat#1863004
ddPCR^TM^ Buffer Control for Probes	Bio-Rad	Cat#1863052
DG32 Automated Droplet Generator Cartridges	Bio-Rad	Cat#1864109
QX200 Droplet Digital PCR System	Bio-Rad	Cat# 1864001
Eppendorf Thermomixer C	Eppendorf	Cat#5382000023
Eppendorf Thermomixer SmartBlock 1.5 mL	Eppendorf	Cat#13687712
EasySep™ Human CD4+ T Cell Isolation Kit	STEMCELL	Cat#17952
EasyEights™ EasySep™ Magnet	STEMCELL	Cat#18103

**Table 1:** HIV-1 multiplex primers and probes

Multiplex assay	Primer/probe	Dye, quencher	Sequence 5′ to 3′	NC_001802 coordinates downloaded 27June19
assay1	3′*pol* F		AGAGATCCACTTTGGAAAGGACCAGCAAA	4466–4494
assay1	3′*pol* probe	FAM, BHQ	T**G**GAA**AG**G**T**GA**A**GG	4502–4515
assay1	3′*pol* R		CACTACTTTTATGTCACTATTATCTTGTATTACTACTGC	4517–4555
assay1	*tat* F		TTTGTTTCATAACAAAAGGCTTAGGCATCTCC	5483–5514
assay1	*tat* probe	FAM, BHQ	**ATG**GCA**G**GA**A**GAAG	5516–5529
assay1	*tat* R		TGAGGAGCTCTTCGTCGCTGTCTCC	5531–5555
	5′ *pol* F		TCCTTTAGCTTCCCTCAGATCACTCT	1787–1812
assay2	5′ *pol* probe	FAM, BHQ	TT**GG**CA**AC**G**A**CC	1813–1824
	5′ *pol* R		TACTGTATCATCTGCTCCTGTATCTAATAGAGCTTC	1859–1894
	*gag* F		GACTAGCGGAGGCTAGAAGGAGAGA	310–334
	*gag* probe	HEX, BHQ	A**TG**GG**T**G**C**GAGA	336–347
	*gag* R		CTAATTCTCCCCCGCTTAATAYTGACG	345–375
assay1&2	*env* F		TGTTCCTTGGGTTCTTGGGAGCAGCAGG	7323–7350
assay1&2	*env* probe	HEX, BHQ	**AG**C**A**CT**ATG**GG	7352–7362
assay1&2	*env* R		GCACTATACCAGACAATAATTGTCTGGCCTGTACC	7384–7418

Bold text indicates locked nucleic acids.

**Table 2:** Primers and probes for the deltaD target in *TRD*

Primer/probe	Multiplex assay	Dye, quencher	Sequence 5′ to 3′	GenBank gene ID 6964 (TRD) coordinates downloaded 27June19
deltaD F	RPP30_deltaD		GCTGGCTGTAATGGGAATGT	18144–18163
deltaD probe	RPP30_deltaD	FAM, BHQ	TGTGAAGATGTCTGTAGCCATCTTAT	18171–18196
deltaD R	RPP30_deltaD		TAATGGCTTGATAAAGATAAGTGATCAT	18201–18228

**Table 3:** Primers and probes for the two targets in *RPP30*

Primer/probe	Multiplex assay	Dye, quencher	Sequence 5′ to 3′	GenBank gene ID 10556 (*RPP30*) coordinates downloaded 24June19
5′*RPP30* F	RPP30_deltaD		GCATATCAGGGTACAGCATAGG	16599–16620
5′*RPP30* probe	RPP30_deltaD	FAM, ZEN/IBFQ	TCTGCTCGTTGTTAGTCACCAGCT	16635–16658
5′*RPP30* R	RPP30_deltaD		CTTCCCTCACGGCATATACTTC	16678–16699
3*′RPP30* F	RPP30_deltaD		GCTGTGTTTGCTCTCTTGATTT	28266–28287
3′*RPP30* probe	RPP30_deltaD	HEX, ZEN/IBFQ	AATGTCTGTGACTGGGTTCTGGCT	28322–28345
3′*RPP30* R	RPP30_deltaD		GGTCTGTCCATGGCATCTTAT	28348–28368

## Materials and equipment

### Equipment

We recommend using a shaking heat block such as the Eppendorf Thermomixer C (Eppendorf Cat#5382000023) during the restriction enzyme step. An alternative is to use a tube rotator inside an incubator.

QX200 Droplet Digital PCR System (Bio-Rad):

This protocol assumes knowledge of the QX200 digital droplet PCR system (droplet generator and reader). Reference manuals are available from the Bio-Rad website: https://www.bio-rad.com/webroot/web/pdf/lsr/literature/10031906.pdf

### Reagents

Commercially prepared HIV negative human genomic DNA is convenient to use for preparing controls (ex. Promega Cat#G3041), but it is not necessary if an equivalent is available.

### Kits

If T cell isolation is to be performed using the EasySep™ Human CD4+ T Cell Isolation Kit (StemCell Cat#17952) prior to DNA extraction, you must also have access to the EasySep™ magnets. The EasyEights™ EasySep™ Magnet is useful for processing multiple samples at a time.

### Data analysis

In order to be able to quantify data about multi-positive populations, .qlp files exported from the Bio-Rad QX200 software must be analyzed in Bio-Rad’s Quanta-Soft Analysis Pro software, which is available for free download (with registration) from https://www.bio-rad.com/en-us/product/qx200-droplet-digital-pcr-system?ID=MPOQQE4VY

See also: Bio-Rad’s Droplet Digital ^TM^ PCR Applications Guide for mathematical details. Page 35 derives the equation for calculating concentration from droplet counts and volume.

https://www.bio-rad.com/webroot/web/pdf/lsr/literature/Bulletin_6407.pdf

All steps in the analysis workflow subsequent to initial analysis in QuantaSoft Analysis Pro can be carried out using MS Excel or a similar spreadsheet program. However, we recommend the use of a script-based software/GUI such as R/Rstudio to make the analysis more reproducible and potentially faster.

#### Example data and analyses

**Excel**: The excel workbook “V3_annotated_example_data.xlsx” contains example data and examples of a spreadsheet-based workflow. The first tab shows an example plate layout following the requirements of the plater package (Hughes, 2016). This format is not strictly required for excel-based analysis but is required if the instructions for analysis in R are followed and may be useful for other data manipulations. The second tab is an orientation to the cluster data that is exported from QuantaSoft. Subsequent tabs depict further steps of the workflow as described below.

**R**: An .RProject directory is included with this protocol with example data and scripts. See the README file in the directory for a description of the contents and how to use it.

## Step-by-step method details

### Tissue lysis: If starting material is cells, not tissue, skip this and proceed to next section

**Timing: 1–2 h hands-on, then 18 h incubation. Begin the protocol in the late afternoon so the 18 h incubation can bescheduled overnight.**

***Note:*** If working with tissues, proceed through this section, if working with cells, skip to the “cell lysis and thawing” section below.

1. When working with tissues, clean forceps by soaking in 70% EtOH for 5 min followed by a rinse in 1**×** PBS or use disposable sterile forceps.
2. Warm complete cell culture medium to 37°C, 5 mL per sample and put 5 mL of warmed medium in a petri dish or well of a 6-well plate. For vaginal, cervical and rectal tissues, we recommend complete RPMI medium: RPMI medium with 10% fetal bovine serum, 1% L-glutamine and 1% pen-strep. Please see the key resources table for catalog numbers for these items. If you are attempting this protocol with a different type of tissue, we a recommend using the same medium as would be used for culture or short-term transport of the tissues.

3. For each sample, label and weigh an empty 1.5–2 mL tube and record the weights.
4. If the tissues are dry frozen:
  a. Get dry ice to store replicate samples if you are working with >1
  b. Using forceps, remove the tissue from the tube and place in the medium to thaw
5. If the tissues are cryopreserved:
  a. Get dry ice to store replicate samples if you are working with >1 at a time.
  b. Place the cryovial in a 37°C water bath and thaw until a small (e.g., 3**×**3**×**3 mm) piece of ice is left.
  c. Using forceps, remove the tissue and place into the warmed medium.
  d. Leave the tissue in the warm medium for 5 min to remove the cryo-preservative.
6. Blot the tissue on a lint-free laboratory wipe to remove excess liquid clinging to the surface.
7. Put the tissue in the pre-weighed tube and weigh the tube again. Calculate the tissue weight by subtracting the weight of the empty tube.
  a. If the tissue is ≥ 7 mg, trim until it is < 7 mg using a sterile scalpel or razor blade. Pieces > 7 mg will likely not digest well with this protocol. Individual samples can be split into multiple tubes and recombined later.
8. Add 180 μL buffer ATL to each tube, making sure that the tissue is submerged in the buffer.
9. Add 20 μL 20 mg/mL proteinase K to the tube.
10. Mix by pulse-vortexing for 15 s. It is important that the proteinase K is well mixed into the solution.
11. Place the tube in the heat block and incubate without shaking at 56°C for **at least 18 h** until the sample is completely lysed.
  a. If possible, check the digestion after a few hours to see how it has progressed. As the tissue digests the original piece will dissolve and no longer be recognizable as a solid chunk. Depending on tissue type, a shorter incubation may be possible for complete digestion/lysis.
12. After 18 h of incubation, gently invert the tube and visually inspect to see if tissue has fully digested.
  a. It is useful to hold the tube up to a dark surface to see if there are remaining light-colored undigested tissue pieces floating in the tube.
  b. If sample is completely dissolved, proceed to genomic DNA (gDNA) isolation.
  c. If sample has not fully lysed (e.g., tissue is still intact, or there is visible material in suspension), repeat proteinase K addition as described above and return samples to heat block. Check after a few hours or up to 18 h (this long incubation can be performed overnight) until the tissue is digested completely. If the tissue does not digest, the piece of tissue may have been too large.
13. Once the tissue is lysed, proceed to gDNA extraction

### Cell thawing and lysis (if starting material is cells, not tissue)

**Timing: 1–2 h depending on number of samples and if isolating CD4**^**+**^**cells**

14. Thaw cryopreserved peripheral blood mononuclear cells ( PBMCs) or CD4^+^ T cells into 37°C complete culture medium.
15. Spin cells for 10 min at 500 **×** *g* to pellet the thawed cells so that the cryopreservative can be removed.
16. Discard supernatant and resuspend cells in an appropriate volume for counting.
17. Count total cells, or, if not already done, isolate CD4^+^ T cells using the EasySep™ Human CD4+ T Cell Isolation Kit (StemCell 17952) or your preferred method, then count the cells.
  a. Counting may be performed manually with a hemocytometer or with an automated cell counter. Once a method is chosen, it should be used for all samples going forward to avoid batch to batch differences.
    i. If cells are counted manually, a minimum of 200 cells should be counted on each side of the hemocytometer to ensure an accurate count.
    ii. If an automated cell counter is used, make sure that the instrument has been calibrated to the company’s specifications and any necessary quality control and cleaning steps have been performed prior to counting the experimental sample.

***Note:*** that while we recommend isolating CD4+ T cells when possible, this method can work on samples in which CD4+ T cell separation is not ideal such as cells frozen without cryopreservation.

18. Transfer up to 2 **×** 10^6^ cells to a 2 mL conical tube (ex. “Eppendorf”). Samples with more cells should be split and processed separately.

***Note:*** Check the centrifuge rating for the tubes and do not spin above this speed as the tubes may crack.

19. Pellet cells by spinning at 2,400 **×** *g* in a benchtop centrifuge for 5 min.
20. Remove supernatant and discard into 10% bleach by pipetting.
21. Using wide-bore filter tips, resuspend the pellet in 100 μL of 3M guanidine HCl
  a. If wide-bore tips are not available, use sterile scissors or a sterile razor blade to cut 2–3mm from the narrow end of a regular bore tip. This will leave a wider orifice like in a commercially sourced wide-bore tip.

**CRITICAL:** Pipette gently using wide-bore filter tips to avoid shearing the DNA.

22. Add 20 μL of 20 mg/mL proteinase K.
23. Invert gently five times to mix thoroughly. It is important to mix gently to avoid shearing the DNA.
24. Incubate at 56°C for 1 h. During this time, put the 6M guanidine isothiocyanate in 37°C water bath or incubator to dissolve any precipitates. Vortex occasionally to help the crystals dissolve.
25. Proceed to gDNA extraction.

### gDNA extraction

**Timing: 30 min for one sample, more time is needed with additional samples**

**CRITICAL:** Dispose of guanidinium salts as chemical waste according to your facility’s rules. Do not mix with bleach, do not pour down drain.

***Note:*** This step includes the initial extraction of gDNA using guanidine salts, precipitation and washing with 70% ethanol. At this step, cell samples will contain 20 μL of Guanidine HCl (GuHcl) and 20 μL of 20 mg/mL proteinase K (Wiegand et al., 2017). Tissue samples will contain 180 μL of buffer ATL and 20 μL of 20 mg/mL proteinase K.

26. Add 400 μL 6M guanidine isothiocyanate to the sample tube.
27. Mix :
  a. For a **cell** sample, mix by pipetting with wide-bore filter tips.
  b. For a **tissue** sample, invert the tube several times (pipette tips may clog).
28. Incubate at 56°C for 10 min.
29. Add 1 mL of 100% molecular grade isopropanol.

***Note:*** Isopropanol should be at about 25°C, cold temperatures will cause more contaminating salts to precipitate out with the DNA.

30. Invert the tube 10 times to mix.
31. Centrifuge for 10 min at the maximum speed (e.g., 21,000 **×** *g*) your tubes are rated for.
32. Pipet off most of the supernatant, leaving ∼100 μL behind.
  a. Dispose of the supernatant according to your facility’s procedures for guanidine.
33. Add 1 mL of 70% ethanol to wash the pellet.
34. Invert the tube 10 times to mix.
35. Centrifuge 1 min at maximum speed.
36. Completely remove the supernatant with a pipette.
37. Spin down briefly with a micro benchtop centrifuge and remove any remaining liquid with a 20 μL pipette tip.

Air dry 5–10 min (lid open) on bench, in fume hood or biosafety cabinet. The pellet may be mostly clear or an opaque white color when it is still saturated with ethanol. Due to the viscosity of the high molecular weight DNA, the precipitated salts may spread into a “smear” on the side of the tube rather than a tight pellet. This is not a problem.

38. Check that the precipitate appears dry and crystalline and that there is no odor of ethanol. If it appears wet and/or there is an odor, continue drying until it meets the criteria above.
39. Add 300 μL buffer EB and vortex 5 s.
40. Incubate at 60°C for 1 h to dissolve the pellet.

***Note:*** The extracted nucleic acid will be high molecular weight at this point in the protocol and will therefore be much more viscous than nucleic acid extracted over a column or with intentional shearing. Avoid pipetting, vortexing, or freezing the sample at this point in the protocol: the goal is to maintain as much intactness as possible. All required fragmentation is achieved through restriction enzyme digestion at the next step. It is not necessary to attempt to quantify the DNA at this point in the protocol, it will likely be inaccurate due to the viscosity of the material.

### Restriction enzyme digestion

**Timing: 20 min hands-on, 19 h incubation**

In this step, the restriction enzyme *Bgl*I (New England Biolabs, Cat# R0143S) is used to digest the high molecular weight nucleic acid isolated in the previous step. This enzyme was specifically chosen because it has cut sites throughout the human genome whereas most proviral genomes do not contain any *Bgl*I cut sites. The high molecular weight extraction followed by this specific enzyme digestion allows controlled fragmentation of the genomic DNA. Instructions for preparing an HIV negative control sample from commercially sourced human genomic DNA are in the preparation section above.

***Note:*** If there are multiple samples to be digested, make a master mix of 10**×** NEBuffer 3.1 and NEB *Bgl*I with the volumes described below and aliquot 48.2 ul into each sample.

41. Bring 10**×** NEBuffer 3.1 to ambient temperature.
  a. If salts have precipitated in the buffer, place in a 37°C incubator or water bath for about 5 min, then vortex, to dissolve the precipitates.
42. Add 35 μL 10**×** NEBuffer 3.1 to the sample (which is already suspended in 300 μL buffer EB).
43. Add 13.2 μL NEB *Bgl*I enzyme (132 units).
44. Invert a few times to mix.
45. Put samples in the Thermomixer C using SmartBlock 1.5 mL with the lowest shaking speed and incubate overnight (16–18 h) at 37°C.

***Note:*** 300 RPM is the lowest shaking speed for all blocks that fit in the Thermomixer C.

**Pause point:** Overnight incubation 16–18 h.

46. After 16–18 h incubation, add 5 μL more *Bgl*I and incubate at 37°C for 1 h in the thermomixer at the lowest speed, which is approximately 300 RPM.

### EtOH precipitation of *Bgl*I-digested samples

47. Get wet ice in a suitably sized ice bucket for the number of samples you have.
48. Put 70% and 100% ethanol on dry ice or in a freezer to cool. These reagents do not need to reach a certain temperature; the cooling is only for making the precipitation more efficient. Salts precipitate more at colder temperatures.
49. Set a heat block for 65°C
50. Add 35 μL of 3M sodium acetate (bringing the solution to 0.3M sodium acetate) to the tubes containing the *Bgl*I-digested samples.
51. Add 766 μL of ice-cold 100% ethanol and mix the solution by inverting.
52. Place the tube on wet ice for 30 min.
53. Centrifuge at maximum speed (e.g., 21,000 **×** *g*) your tubes are rated for 10 min.
54. Carefully pipette off the supernatant down to 50–100 μL residual volume.
55. Add 1 mL of 70% ethanol and centrifuge at maximum speed for 2 min.
56. Spin down briefly with a micro benchtop centrifuge and remove any remaining liquid with a 20 μL pipette tip.
57. Air dry 5–10 min (lid open)

**CRITICAL:** The pellet of precipitated nucleic acid must be free of any remaining alcohol.

58. Add 50 μL of buffer EB to the pellet and allow it to dissolve 1 h at 65°C, then leave up to overnight at room temperature, about 25°C.
  a. If the gDNA is still viscous, repeat the restriction enzyme digestion and EtOH precipitation.

### Quantify concentration and dilute

**Timing: Depends on number of samples, at least 15 min**

59. Measure the concentration of the gDNA with a NanoDrop or other spectrophotometer.
60. If the gDNA is >200 ng/μL and <300 ng/ul after the EtOH precipitation, dilute with Qiagen Buffer EB to a maximum of 200 ng/μL for the HIV assays. If the gDNA is >300 ng/ul, repeat *Bgl*I digestion.
61. Prepare at least 20 μL of 1:100 diluted gDNA in Qiagen Buffer EB or molecular grade water. This will be used for the two replicate wells of the reference assay.

**CRITICAL:** The values from the reference assay are used to make inferences about the concentrations in the HIV assay wells. Therefore, it is important to prepare the 1:100 dilution accurately. To minimize error, use a minimum of 5 μL stock template and 495 μL buffer EB.

62. Store the gDNA at 4°C until ready to proceed with ddPCR.

### Add reagents to plates and generate droplets

**Timing: Depends on number of samples, at least 20 min**

63. Get out two new twin-tec ddPCR 96–well plates, one for the master mix and DNA and one to receive the droplets once they are formed in the droplet generator.
64. Each well will receive a total of 22 μL before droplet generation: 17 μL of master mix and 5 μL of template. First prepare the master mix:
  a. For a single well, the master mix consists of 11 μL of 2**×** supermix and 3 μL of the assay pool (either the assay 1, assay 2 or RPP30_deltaD assay pools described above) and 3 μL of molecular grade water. Prepare enough master mix for the number of wells you are running plus 10% overage.
65. Add 17 μL of master mix (supermix + assay pool + water) per well
  a. Fill the plate column-wise to save on droplet generator consumables
  b. If there are any columns that don’t have master mix in all 8 wells, add 22 μL of 2**×** ddPCR Buffer Control for Probes to the empty wells in those columns.
  c. *Gently* tap the plate on the bench to bring reagents to the bottom of the wells.
66. Seal the plate with an adhesive plate seal to prevent contamination if you are not immediately adding template DNA or need to move to the plate to another area.

### Add template DNA and plasmid control samples to plate

67. Add 5 μL template to the wells according to the plate layout: the HIV assays should be run in triplicate and the reference assays run in duplicate (see NOTE). We suggest that for each column, wells A, B and C are used for HIV assay1, D, E and F are used for HIV assay2 and wells G and H are used for the reference assay. An example plate layout is available in spreadsheet form in the supplemental folder **“**Data S1**”.** The file is called **“05162019_PTID1031_plate_layout_CL.csv**”
  ***Note:*** If the sample has very low levels of integrated provirus, it may be necessary to survey a larger volume of sample to pick up this rare signal. If this is suspected, we suggest increasing the number of replicates of the HIV assay wells so that a larger amount of DNA can be assayed.
  a. Add **undiluted** gDNA to the HIV assay wells.
    **CRITICAL:** DNA used for the ddPCR template should have a maximum concentration of 200 ng/μL. A higher concentration will interfere with droplet formation.
  b. Add **diluted template** to the RRP30_deltaD assay wells: undiluted template will likely lead to an oversaturated signal (all droplets positive) and an underestimate of DNA shearing.
    i. For experimental (derived from a study participant as opposed to a plasmid control or other artificially prepared sample) samples, dilute the gDNA 1:100 with Qiagen Buffer EB.
    ii. For the plasmid/HIV-negative gating controls, dilute 1:2 with Qiagen Buffer EB.
      ***Note:*** This dilution may need to be adjusted due to batch to batch variation in plasmid controls or degradation of the controls over time. The goal is to have enough positive droplets for clear populations and this will vary from batch to batch of controls.

68. Apply the foil seal to the plate with the auto sealer and invert the plate several times to thoroughly mix the master mix and DNA in the plate.
69. Spin down the plate to bring all liquid to the bottom of the wells.

**CRITICAL:** Check for bubbles on the bottom of the wells. Spin again if you see any. Bubbles on top are ok. Bubbles on the bottom will interfere with droplet generation by the autoDG.

### Generate droplets

**Timing: Up to 35 min, or less if entire plate is not used**

***Note:*** See the autoDG manual for details on use: https://www.bio-rad.com/webroot/web/pdf/lsr/literature/10043138.pdf

70. Assemble the autoDG (droplet generator) with cartridge, tips, template plate and empty plate (where the newly generated droplets will go) in their appropriate locations.
71. Configure the wells on the autoDG and initiate droplet generation.
72. When the droplets are done, heat seal the droplet plate with a pierce-able foil seal with auto sealer and dispose of the plate that held the master mix and DNA.
73. Observe wells for any abnormalities in droplet formation: there should be a uniform opaque layer at the top of each well.

### Thermal cycling (PCR)

**Timing: about 2.5 h**

74. Transfer the droplets plate to the thermal-cycler and run the ddPCR protocol. The ramp rate should be 2°C for all steps. The run volume is 40 μL per well.

**CRITICAL:** The plate must be completely sealed around all the edges to avoid evaporation during PCR cycling.

**Table:** 

PCR cycling conditions			
Steps	Temperature	Time	Cycles
Initial enzyme activation	95°C	10 m	1
Denature	94°C	30 s	60 cycles
Anneal and extend	60°C	60 s	
Enzyme deactivation	98°C	10 m	1
Hold	4°C	forever

**Pause point:** After the cycling is complete, the plate can be stored overnight (12–18 h) at 4°C before reading the droplets.

### Read droplets

**Timing: 1–2 min per well, 2 h for an entire 96-well plate.**

75. When the PCR reaction is done, take the plate to the droplet reader, put the plate in the holder, and clamp it down.
76. Fill out the plate info in the QuantaSoft software.
  a. Highlight the wells that you are going to read.
  b. Supermix is ddPCR supermix for probes (no dUTP).
  c. Assign channel 1 and channel 2 to unknowns.
77. Click run plate, then save the template file.
  a. Choose the FAM/HEX dye set.
78. Wait until the first well starts to make sure there are no issues initiating the reading.

## Expected outcomes

If the initial input is about 2**×**10^6^ PBMC or T cells, the maximum expected total output of extracted nucleic acid is approximately 10 ug (50 μL of 200 ng/μL). The expected droplet count is between 15,000 and 20,000 droplets per well. We recommend excluding any wells with fewer than 10,000 droplets due to reduced accuracy.

## Quantification and statistical analysis

### Overview

The main goal of this analysis is to convert droplet counts, which can be exported directly from QuantaSoft AP, to the number of copies of template that was positive for all three HIV targets (i.e., potentially intact HIV provirus), normalized to total cells or total T cells (e.g., X copies per 1E6 T cells).

Definitions:

**Target**: The sequence of interest that is targeted by a set of primers and a corresponding probe (e.g., gag or env).

**Cluster**: A group of droplets positive for a particular combination of targets. For example, the “triple positive” cluster is positive for all three targets in an assay.

**Sample**: The source of template DNA. Each sample is measured in two HIV assays (three replicate wells each) and a reference assay (two replicate wells).

**Merged counts**: Summed droplet counts across the replicate wells for a single sample, accounting for cluster membership.

**Total merged count:** The total number of droplets in a merged well: all the droplets, regardless of cluster membership.

**Negative droplets:** Droplets that are negative for all targets OR droplets that are negative for the cluster of interest.

Set-up: For each assay (the two HIV assays and the reference assay):

• “Merge” the replicate wells by adding up the droplet counts from replicate wells as if they were in a single big well.

For the reference assay only:

• Correct the concentration based on the dilution of the template.
• Correct the concentration for diploidy.

Relate the reference targets to the HIV targets and normalize.

• Divide the concentration of each HIV cluster by the concentration of total cells or T cells.
• Normalize to millions of total cells or total T cells.

Calculate the five-target estimate (“5-TE”)

• Equations that will be used:

**Equation A**: Convert droplet counts to concentration in copies per μL

$$Copies\;per\;\text{μ}L=-\ln\;\left(\frac{negative\;droplets}{total\;droplets}\right)÷droplet\;volume$$

**Equation B**: Calculate the DNA shearing index (DSI):

$$DSI=\;\frac{average\;single}{average\;single+RPP30\;double\;positive}$$

**Equation C:** Correct observed triple positive copies for DNA shearing

$$Corrected=\;\frac{observed}{1-DSI}$$

**Equation D**: Calculate the probability that a droplet is positive for all three targets

$$p\left(triple\;positive\right)=\;\frac{triple\;positive\;concentration}{total\;HIV\;concentration}$$

**Equation E**: Calculate the probability that a droplet is positive for all targets in assay1 *and* assay2:

$$p\left(5\;target\;positive\right)=p\left(triple\;positive_{assay1}\right)\times p\left(triple\;positive_{assay2}\right)$$

**Equation F:** Determine the five target estimate (the estimated number of droplets containing template with all 5 targets)

$$p\left(5\;target\;positive\right)=p\left(5\;target\;positive\right)\times \frac{H1+H2}{2}$$

Where:

*H1* = total HIV copies per 10^6^ T cells by assay1

*H2 =* total HIV copies per 10^6^ T cells by assay2

**Example data: Excel users,** please see the Excel file “V3_annotated_example_data.xlsx” in supplemental folder Data S2 for further description of analyzing the data in a spreadsheet program. **R users**, please see the .RProject directory in supplemental Data S2 for examples of using scripts to analyze the data.

### Export cluster data from QuantaSoft AP

1. Open the .qlp file in QuantaSoft AP (“QSAP”) and assign targets and assays:
  a. Experiment type: “Direct Quantification (DQ)”
  b. Highlight all the HIV assay1 wells and in the plate editor tab, under “Assay Information” set these wells as “Amplitude multiplex” and assign the targets and dyes specific to assay1
  c. Name: <Enter sample name> (optional)
  d. Type: “Unknown”
  e. Click the “ – “ sign to the left of the row with the Target Name “Target 4” to remove this row. There are only 3 targets per assay so the fourth slot is not needed.

**Table:** Make the following assignments for assay1 wells:

Target name	Target type	Signal Ch1	Signal Ch2
3′pol	Unkn	FAM Lo	None
Tat	Unkn	FAM Hi	None
Env	Unkn	None	HEX Hi
*Target 4*	*Unkn*	*None*	*HEX Lo*

**Table:** assay2 wells:

Target name	Target type	Signal Ch1	Signal Ch2
5′pol	Unkn	FAM Hi	None
Gag	Unkn	None	HEX Lo
Env	Unkn	None	HEX Hi
*Target 4*	*Unkn*	*FAM Lo*	*none*

**Table:** RPP30_deltaD assay wells:

Target name	Target type	Signal Ch1	Signal Ch2
deltaD	Unkn	FAM Lo	None
5′*RPP30*	Unkn	FAM Hi	None
3′*RPP30*	Unkn	None	HEX Hi
*Target 4*	*Unkn*	*None*	*HEX Lo*

2. Repeat previous step for assay2 wells and the reference assay wells
3. In the Droplets tab, check if any wells had <10k droplets. These should be excluded from the analysis later on, but it is good to check now in case there are obvious patterns such as all wells in one row having a low droplet number. This could indicate a problem with the droplet generator or a pipetting problem.
4. Apply auto-gates to the plasmid control wells
5. Apply the plasmid-based gates to the sample wells
6. Highlight all wells and export the Well Data in csv format.

***Note:*** .csv format is required if you plan to use R for analyses. Excel is ok if analysis will be carried out in a spreadsheet program.

7. Go to “Analysis Tools” and “Export Cluster Data”
  a. Target names assigned in the Plate Editor will *not* appear in the cluster data, there will only be “Target 1”, “Target 2″and “Target 3” as column names. These names are assigned to targets in the following order: FAM Lo, FAM Hi, HEX Lo, HEX Hi.

**Table:** 

Assay	Target 1	Target 2	Target 3
assay1	3′pol (FAM Lo)	tat (FAM Hi)	env (HEX Hi)
assay2	5′pol (FAM Hi)	gag (HEX Lo)	env (HEX Hi)
RPP30_deltaD	deltaD (FAM Lo)	5′*RPP30* (FAM Hi)	3′*RPP30* (HEX Hi)
  b. Sample names assigned in the Plate Editor will *not* appear in the cluster data, samples can be identified by plate location (ex. “A01”). The R-based analysis example uses the package “plater” to merge meta-data with the output from QuantaSoft AP, which is one way to add Sample IDs to the results. For use with the R scripts, meta-data must be prepared in the format shown in the “example_plate_layout” tab of the “V3_annotated_example_data.xlsx” file in supplmental folder Data S1.

### Analysis of data after export from QSAP

***Note:*** The following instructions refer to examples in the Excel example data. In the example spreadsheet, plain text represents data exported from QuantaSoft or carried over from a previous tab. Yellow highlighted data indicates new calculations that have been done in the worksheet. There are 12 tabs in **“V3_annotated_example_data.xlsx”**. The protocol below will reference the tab where an example can be found. Formulas are included in the spreadsheet cells to guide the calculation.

R users should review the README file provided with the supplemental R project file (Data S2) (Wickham, 2017; Hughes, 2016; Muller, 2020; Lai, 2020; RStudio, 2015). The scripts also contain comments detailing the steps and functions. The steps are generally the same as those described below for the Excel workflow but modified for efficiency (steps are performed on all samples at once) and data objects are formatted for manipulation using tidyverse (Wickham, 2017) functions.

### Set up cluster data

8. Confirm plate layout (tab 1)
9. Open exported cluster data. (tabs 2–4).
10. Make a fake data set that includes all possible clusters for every sample. (Excel example data, tab 5) This step is necessary because QSAP only includes rows for clusters that were present for that sample (i.e., it will not list a cluster and indicate that there were zero droplets, there will simply be a row missing, tab 3, but this information is necessary).
11. Use the Query tool in Excel to perform an anti-join to see which rows in the “all possible clusters” created in the previous step data are missing from the real data. The anti-join merges the data so that the resulting table shows all rows present in one data set and absent in the other data set. (tab 6) The exact steps required to perform the anti-join will depend on your version of excel. See the following link for a tutorial on anti-joins with the Query tool: https://docs.microsoft.com/en-us/power-query/merge-queries-left-anti
12. Add the missing clusters (with zero as the count) to the exported data (tab 7) by copying the results of the anti-join and pasting them to the bottom of the data set that was exported from QuantaSoft AP (in this example, we use the data from tab 4). After pasting the “missing” values, sort the results by Well to put all the rows belonging to the same Well in consecutive rows of the data set (tab 7).
13. Add meta data including Assay and unique sample identifiers that will be needed for sorting data (tab 8).

Calculate HIV triple positive copies per μL from droplet counts

14. Tab 9: This example will use assay1 results from a single sample. The same steps should be performed for the rest of the samples and for assay2.
  a. Section A shows the three replicate wells of assay1 for sample_01. There are 8 rows per well, each representing a different cluster of targets.
    i. Rename the column headers “Target 1”, “Target 2” and “Target 3” with the actual HIV target names for assay1.
  b. Section B: Calculate the total droplets in each well, the total triple positive droplets and NON-triple positive droplets in each replicate well.
    i. Confirm that each well meets the required threshold of at least 10,000 droplets total. If not, exclude that well.
  c. Section C:
    i. “Merge” the counts from the 3 replicate wells by adding the counts together from section B.
    ii. Use the total merged and non-triple positive counts and the known droplet volume to calculate copies per μL of triple positive droplets (**Equation A).**
      **This is the concentration of droplets containing potentially intact provirus. NOTE:** See Bio-Rad’s Droplet Digital ^TM^ PCR Applications Guide, page 35 for mathematical details.
  d. Repeat the steps described in Sections A-C for assay2.

### Calculate copies per μL for reference assay clusters

15. Section D shows the reference assay wells for sample_01. These wells received the same template as the wells in Section A but at a 1:100 dilution.
  a. Add a name for each cluster (section D column H). The cluster name references the targets that make up that cluster, which are indicated in columns B, C and D.
16. Section E: Calculate the merged counts of the clusters across the replicate wells
17. Section F and G: Calculate copies per μL for droplets containing deltaD (alone or with another target) and for droplets containing 5′*RPP30* (**Equation A).**

***Alternatives:*** These concentrations are also available from the “well data” from QuantaSoft AP. “Well data” results show the concentration of each target regardless of whether it was in a cluster with any other target.

### Calculate the total amount of HIV present in any type of cluster

18. Add Cluster names as was done for the reference assay in tab 9 section D. This is shown for assay1 data in tab 10 section H.
19. Sum the counts for each cluster and for all droplets to get merged counts (tab 10 section I).
20. Subtract the merged count for each cluster from the total merged count to get the number of droplets NOT positive for your cluster of interest (tab 10 section I column N).
21. Using the known droplet volume and **Equation A**, calculate the concentration in copies per μL for each cluster (tab 10 section I column P).
22. Calculate the concentration of HIV in any form (i.e., a target alone or in combination with any other target) by summing the all cluster concentrations *except the negative cluster*). See tab 10 section J. **This is the total concentration of any HIV.** This value can be compared to the triple positive results obtained in tab 9 section C.

### Normalize to cell counts

After following the steps above, we have concentrations in copies per μL for all clusters, including the triple positive cluster. Next, we will correct the reference target concentrations for dilution and diploidy, then use those corrected concentrations to normalize the HIV concentrations to copies per million T cells.

23. Multiply the reference gene concentrations by the dilution factor (100). In the reference gene assay, the template is 100**×** more dilute than the template used in the corresponding HIV assay wells. The concentrations therefore need to be multiplied by this dilution factor (100) to allow us to estimate how many cells-worth of DNA was used in the HIV assay wells (tab 11 section K).
24. Divide the reference gene concentrations by 2 to account for diploidy (every cell has two copies of the reference genes; tab 11 section K).
25. Tab 11 section K columns E and F show the values after making the dilution and diploidy corrections. **These are the concentrations of cells per μL and non-T cell per μL used in the HIV assays.**
26. Calculate the number of T cells per μL. 5′*RPP30* represents the number of cells and deltaD represents the number of non-T cells. To get number of T cells, we subtract: total cells – nonT cells = T cells (tab 11 section K column G).

**CRITICAL:** All T cell/μL counts should be >0, otherwise it means that there were more non-T cells that total cells, which could indicate an error. If T cells were isolated prior to DNA extraction, there will be little (or no) DNA from non-T cells (therefore a low concentration of deltaD).

### Correct for DNA shearing

The next step is to correct the observed concentration of triple positive droplets per million T cells for incidental DNA shearing with the DNA Shearing Index (DSI). The DSI is the probability that DNA template was sheared. The reference assay contains two targets in the *RPP30* gene: 5′*RPP30* and 3′*RPP30.* The distance between these targets is approximately the same length as the HIV genome and the *Bgl*I enzyme does not have cut sites in this region. When both *RPP30* targets occur in the same droplet, we can assume that they were on the same piece of template, i.e., the template was not sheared. If one of these targets occurs in a droplet without the other, we know that the DNA was sheared. We can then use the amount of shearing in this known fragment of genomic DNA as a proxy measurement for shearing in general, and apply this probability to the normalized triple positive HIV concentrations (Levy et al., 2021).

27. Use the counts of *RPP30* double and single positive droplets to calculate the DSI using Equation B (tab 11 section M, column F):
28. Using the observed triple positive copies per million cells calculated previously, we divide by 1-DSI to estimate a corrected value that accounts for shearing that reduced the number of triple positive droplets, thus artificially lowering the concentration **(Equation C).** Note that it is only possible to do this correction on the triple positive cluster.

### Calculate 5-target estimate (“5TE”)

Using the values we have calculated above for the total HIV copies per 1E6 T cells and the triple positive copies per 1E6 T cells for each of the two HIV triplex assays, we can calculate the probability that a template is positive for all three targets in the assay using **Equation D,** and the probability that a template is positive for all three targets in assay1 AND all three targets in assay2 with **Equation E.**

Finally, we estimate the actual number of copies that are likely positive for all 5 targets by multiplying the probability by the average total copies per 1E6 cells calculated for the two HIV assays using **Equation F.**

29. Calculate the probability of triple positive for assay1 and assay2 using **Equation D** (tab 12 section N columns F and G).
30. Calculate the average total HIV detected for assay1 and assay2 (tab 12 section N column H)
31. Calculate the “five target estimate” (“5TE”) using **Equation F** (tab 12 section N column I).

## Limitations

The degree of mechanical shearing of the DNA will depend on initial sample quality and preservation. In our experience, tissue samples have more shearing than cell samples.

In samples from volunteers on anti-retroviral treatment, the amount of integrated virus may be very low. A higher number of replicate wells may be needed to measure enough DNA to get a positive signal.

## Troubleshooting

### Problem 1

DNA is viscous and difficult to pipette after extraction or precipitation (Step-by-Step Method Details step 58).

### Potential solution

Use fewer cells or bring the volume of extracted DNA to 300 μL with Qiagen buffer EB and repeat the 18hr *Bgl*I digestion, followed by precipitation.

### Problem 2

Very few or zero droplets form during droplet generation (Step-by-Step Method Details after step 98).

### Potential solution

The template DNA may be too viscous for droplet formation. Repeat the restriction digestion and precipitation.

### Problem 3

Well volume is reduced after thermal-cycling (Step-by-Step Method Details step 78).

### Potential solution

Adjust heat-sealing parameters to ensure a complete seal, check the plate sealer for malfunction.

### Problem 4

There are no triple positive droplets present before shearing correction (Quantification and Statistical Analysis step 26).

### Potential solution

If intact HIV proviral DNA is expected from the sample, there may be no triple positive droplets if the DNA was sheared too much. Check the DSI and the double positive droplet counts for the *RPP30* targets. Another possibility is that the HIV reservoir from which the sample was derived is very small and more DNA needs to be surveyed to detect intact provirus.

### Problem 5

All droplets are positive for a particular target so it is not possible to use the provided equations to calculate target concentrations (Step-by-Step Method Details, after step 78).

This is not likely to occur with HIV targets in human samples but could occur with plasmid or other control samples and could occur when the reference assay is used on improperly diluted human samples.

### Potential solution

All positive droplets are an indication that the target concentration of the input sample is too high. This could occur if the input was not diluted properly or if there is contamination of the template sample. If contamination (for example, of template sample with plasmid) is suspected, first test the PCR reagents, including water, for contamination by running samples without template added to the PCR mixture. If the reagents are contaminated, these “no template” samples will still amplify. If this occurs, replace the contaminated reagents. If the PCR reagents are not contaminated, test several dilutions of the template in case it was not diluted enough. If the dilution factor is not the problem, review lab protocols for template and plasmid containment and prepare new template.

## Resource availability

### Lead contact

Further information and requests for resources and reagents should be directed to and will be fulfilled by the lead contact, Florian Hladik (florian@uw.edu).

### Materials availability

The plasmid sequences used for gating controls are available from Addgene with the following identifiers: 167347, Seattle_IPDA_control_1_001; 167348, Seattle_IPDA_control_6_002; 167349, Seattle_IPDA_control_9_003; 167350, Seattle_IPDA_control_14_004; 167351, Seattle_IPDA_control_17_005; 167352, Seattle_IPDA_control_29_006; 167353, Seattle_IPDA_control_32_007.

## Data Availability

An annotated example data set accompanies this protocol. These data are a subset of the UW_CFAR_KINETICS cohort that was analyzed for our related publication: Levy et al., A highly multiplexed droplet digital PCR assay to measure the intact HIV-1 proviral reservoir, Cell Reports Medicine (2021), https://doi.org/10.1016/j.xcrm.2021.100243. These data are from participant samples that were obtained with approval from the University of Washington Institutional Review Board. In addition to the example data, we provide R code and a step-by-step spreadsheet-based workflow for data analysis in the supplementary documents to this manuscript.

## Supplemental information

Document S1. Figure S1

Data S1. Data analysis example (spreadsheet method), related to quantification and statistical analysis steps 8–31

Data S2. Data analysis example (R method), related to quantification and statistical analysis steps 8–31
