# Supplementary material for: HIV reservoir quantification by five-target multiplex droplet digital PCR
Source: STAR Protoc. 2021 Oct 11;2(4):100885. doi: 10.1016/j.xpro.2021.100885 (PMC8517383; doi:10.1016/j.xpro.2021.100885)
Supplement: Document S1. Figure S1 [file mmc1.pdf]

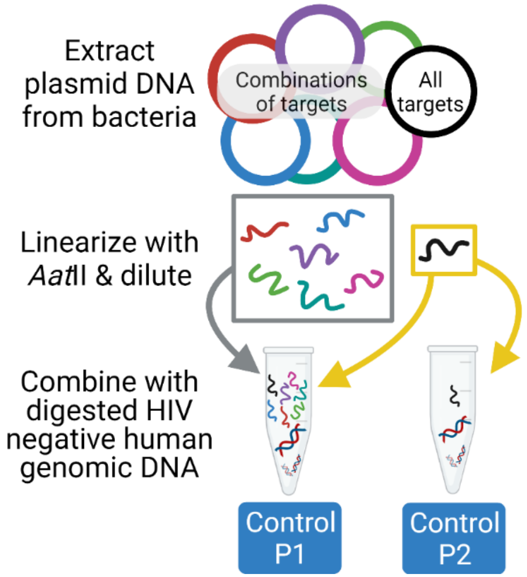

Figure S1: Graphical representation of controls preparation, related to section “Prepare Plasmid Controls”, step 8.
