# Supplementary material for: HIV reservoir quantification by five-target multiplex droplet digital PCR
Source: STAR Protoc. 2021 Oct 11;2(4):100885. doi: 10.1016/j.xpro.2021.100885 (PMC8517383; doi:10.1016/j.xpro.2021.100885)
Supplement: Data S2. Data analysis example (R method), related to quantification and statistical analysis steps 8–31 [file mmc3.zip › data_S2_R_analysis_example_workflow/R_data_analysis_examples_README.html]

R data analysis examples README


# R data analysis examples README

#### Claire Levy

#### Jun 08 11:03:44 2021

## New R users

Please see this Rstudio page for resources for getting started with R/Rstudio.

## Required packages: all packages are available from CRAN

- tidyverse
- plater
- arrangements
- here

## Optional packages:

For making droplet plots outside of QuantaSoft AP: ddpcr

---

## Rstudio Project

We have compiled the scripts and example data into an RStudio Project to allow the use of relative (versus absolute) paths to files and folders. This way it is not necessary to customize the file paths to the file structure on your computer or network.

### Steps to open the project and a script

1. Make sure you have R/Rstudio installed on your computer
2. Un-zip the "R\_data\_analysis\_examples" folder
3. Click on the "/005-R\_data\_analysis\_examples.Rproj". This will open an instance of Rstudio. You may need to direct your operating system to open this type of file with Rstudio.
4. Use File > Open File to navigate to the location where you un-zipped the project folder and open the script, **002-multi\_plate\_analysis\_example.R**. Alternatively, click the Files tab in the Rstudio in the Files/Plots/Packages/Help/Viewer pane and navigate to the scripts.

See the "Working with Projects: Opening Projects" section of this tutorial for more details about working with pre-existing Rstudio projects.

## This project folder contains three sub-folders:

### 001-scripts

### 002-input\_data

### 003-output\_data

---

### 001-scripts: This folder contains three R scripts

**001-multi\_plate\_analysis\_helpers\_and\_libraries.R**

This script contains functions for streamlining data manipulations in the other two scripts. It does not need to be called directly, there are lines of code in the other two scripts that will source this one.

**002-multi\_plate\_analysis\_example.R**

The purpose of this script is to convert raw cluster data from QuantaSoft AP and a researcher-completed plate layout document into a data set containing cluster copies per 1E6 cells or per 1E6 T cells. The "multi\_plate" part of the file name means that this script can analyze data from more than one plate of a results at a time. The motivation for this functionality is that many projects involve the analysis of multiple ddPCR plates of results (instead of just one plate) and it is often desirable for those data to all be analyzed in the same way and for the results to be compiled at the end. The multiple data sets are read into R as a list, so if there is only one plate, this script can still be used, the list objects will still exist but with only a single element. At the end of this script, the normalized results are written out as a .csv document to the 003-output\_data folder one level up from this script.

**003-5TE\_calculations.R**

The purpose of this script is to take the normalized results generated by the 002-multi\_plate\_analysis\_example.R script and calculate the estimated number of intact copies per 1E6 T cells that are positive for all 5 HIV targets. At the end of the script, the results are written out as a .csv document to the 003-output\_data folder one level up from this script.

---

### 002-input\_data

This folder contains example data from three plates, each representing results from a single participant over multiple time points. For each experiment run there are three files: plate\_layout, ClusterData and well\_data. The plate layout is not associated with QuantaSoftAP, it is there for the researcher to add meta data to the analysis. Although sample names can be input into QuantaSoft when the plate is read, users may find it more convenient to incorporate those into the plate\_layout instead, along with any other meta data of interest such as blood draw date, patient identifier etc. QuantaSoft data is connected to the plate\_layout via well ID, which is present both in the exported QuantaSoft data and the plate\_layout information.

**Getting the raw data**

ClusterData and well\_data can be exported directly from QuantaSoft AP after cluster gates are drawn. See the QuantaSoftAP manual for instructions.

**Keeping track of data from multiple plates**

We recommend including a plate specific identifier in each file name to help keep track of the data from each run. In the example data, every file associated with the experiment run on 2019-05-16 from particpant PTID1031 is prepended with "05162019\_PTID1031". These identifying strings can be extracted from the file names and used in the analysis for grouping data. **002-multi\_plate\_analysis\_example.R** uses a regular expression to capture this string: \d{8}\_PTID\d{4}. The regular expression may need to be adjusted to capture your specific identifier.

---

### 003-output\_data

This folder is the destination for the results of running the two analysis scripts. **002-multi\_plate\_analysis\_example.R** uses a function to point to this location but in **003-5TE\_calculations.R** the file path is specified. This folder contains two example output files from the **002-multi\_plate\_analysis\_example.R** and **003-5TE\_calculations.R**. Each time these scripts are run completely, the results will be saved in this folder and the file will be appended with the date that they were generated.

---

### Workflow

Begin with **002-multi\_plate\_analysis\_example.R**. At the top of this script, **001-multi\_plate\_analysis\_helpers\_and\_libraries.R** is sourced.

Assign your expected number of samples to the "EXPECTED\_SAMPLES" variable at the top of the script (for the example data, there are 36). This variable will be used later to check that every sample is accounted for. The analysis is initiated by reading in raw data that was exported from QuantaSoftAP and a plate layout defining meta data for the samples.

Continue stepping through the script interactively. At the end of the script, results are written out as a .csv file to the 003-output-data folder.

The **003-5TE\_calculations.R** script sources **002-multi\_plate\_analysis\_example.R**, so it is not necessary to read in the results from the .csv that was written out by the previous script or to read in new raw data from QuantaSoft AP.

If droplet plots other than those created by QuantaSoft AP are desired, the package ddpcr provides functions for this purpose.
